# Supplementary material for: A peptide encoded by the circular form of the SHPRH gene induces apoptosis in neuroblastoma cells
Source: PeerJ. 2024 Jan 23;12:e16806. doi: 10.7717/peerj.16806 (PMC10812589; doi:10.7717/peerj.16806)
Supplement: Supplemental Information 1 [file peerj-12-16806-s001.docx]

PCR Primer Sequences

| **Target** | **Forward Primer (5'-3')** | **Reverse Primer (5'-3')** |
| --- | --- | --- |
| circSHPRH | CTGAAAACTGCTGAGAGAAGGG | TTGCCACGTTGAGAAAACGA |
| Bcl-2 | GAGGATTGTGGCCTTCTTTG | GTTCCACAAAGGCATCCCAG |
| Caspase-3 | GTGGAACTGACGATGATATGGC | CGCAAAGTGACTGGATGAACC |
| NFKBIA | GGCTGAAAGAACATGGACTTG | GTACACCATTTACAGGAGGG |
| circSHPRH-146aa | CAGCTTAGAGATCCAGGGGC | TGCTGAAAGGTTCTCCTGAAA |
| GAPDH | TGCATCCTGCACCAA | TCCACGATGCCATTG |

Note: Forward Primer (F) is in the direction of 5' to 3', and Reverse Primer (R) is also given in the direction of 5' to 3'.
